# Pancreatic CT perfusion: quantitative meta-analysis of disease discrimination, protocol development, and effect of CT parameters

**DOI:** 10.1186/s13244-023-01471-0

**Published:** 2023-07-21

**Authors:** Stephan Skornitzke, Neha Vats, Philipp Mayer, Hans-Ulrich Kauczor, Wolfram Stiller

**Affiliations:** Diagnostic and Interventional Radiology (DIR), Heidelberg University Hospital, Im Neuenheimer Feld 420, 69120 Heidelberg, Germany

**Keywords:** X-ray computed tomography, Perfusion imaging, Pancreas

## Abstract

**Background:**

This study provides a quantitative meta-analysis of pancreatic CT perfusion studies, investigating choice of study parameters, ability for quantitative discrimination of pancreatic diseases, and influence of acquisition and reconstruction parameters on reported results.

**Methods:**

Based on a PubMed search with key terms ‘pancreas’ or ‘pancreatic,’ ‘dynamic’ or ‘perfusion,’ and ‘computed tomography’ or ‘CT,’ 491 articles published between 1982 and 2020 were screened for inclusion in the study. Inclusion criteria were: reported original data, human subjects, five or more datasets, measurements of pancreas or pancreatic pathologies, and reported quantitative perfusion parameters. Study parameters and reported quantitative measurements were extracted, and heterogeneity of study parameters and trends over time are analyzed. Pooled data were tested with weighted ANOVA and ANCOVA models for differences in perfusion results between normal pancreas, pancreatitis, PDAC (pancreatic ductal adenocarcinoma), and non-PDAC (e.g., neuroendocrine tumors, insulinomas) and based on study parameters.

**Results:**

Reported acquisition parameters were heterogeneous, except for contrast agent amount and injection rate. Tube potential and slice thickness decreased, whereas tube current time product and scan coverage increased over time. Blood flow and blood volume showed significant differences between pathologies (both *p* < 0.001), unlike permeability (*p* = 0.11). Study parameters showed a significant effect on reported quantitative measurements (*p* < 0.05).

**Conclusions:**

Significant differences in perfusion measurements between pathologies could be shown for pooled data despite observed heterogeneity in study parameters. Statistical analysis indicates most influential parameters for future optimization and standardization of acquisition protocols.

**Critical relevance statement:**

Quantitative CT perfusion enables differentiation of pancreatic pathologies despite the heterogeneity of study parameters in current clinical practice.

**Graphical abstract:**

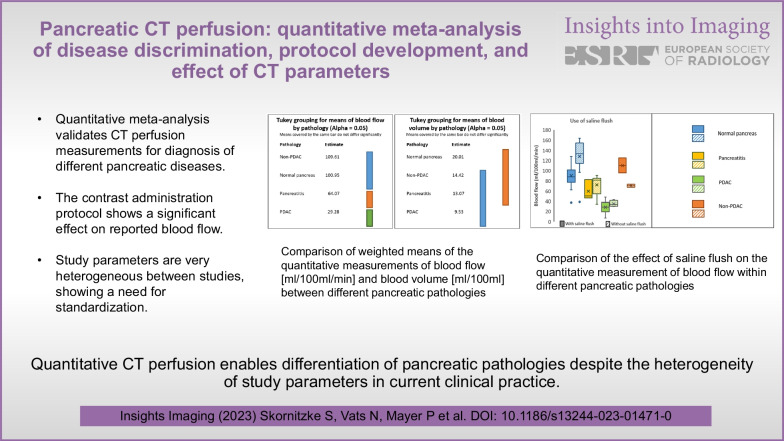

## Key points

1. Quantitative meta-analysis validates CT perfusion measurements for diagnosis of different pancreatic diseases.
2. The contrast administration protocol shows a significant effect on reported blood flow.
3. Study parameters are very heterogeneous between studies, showing a need for standardization

## Background

Dynamic CT perfusion is a functional imaging technique that enables measurement of physiological parameters of blood flow for the assessment of diseases that influence tissue perfusion. Early applications of CT perfusion focused on the brain [1], but some early studies also performed dynamic contrast-enhanced CT acquisitions of the pancreas without calculating quantitative parameters [2, 3]. Since then, new mathematical models of CT perfusion have been developed [4, 5] and applied to the pancreas [6, 7]. Studies have shown that quantitative measurements of physiological perfusion parameters by means of CT perfusion can aid in the detection of pancreatic ductal adenocarcinoma (PDAC) [8], even in difficult cases that appear isodense to the surrounding tissue on conventional CT images [9–11], and allow therapy response assessment for treatment of PDAC [12]. Furthermore, CT perfusion can be used to assess the severity of pancreatitis [13] as well as for the distinction of pancreatic diseases [14], i.e., to improve the difficult differentiation between mass-forming pancreatitis and PDAC [10, 15].

In view of the current role of imaging modalities in the workup of these pancreatic diseases, the German S3 guideline recommends preoperative liver magnetic resonance imaging (MRI) with diffusion-weighted imaging (DWI) in every patient with potentially resectable PDAC [16] for the reason of MRI being superior for the detection and characterization of liver lesions that are undetectable or indeterminate on CT [17, 18]. In 2017 in turn, a systematic review and meta-analysis rated transabdominal ultrasound (US) equivalent to CT and MRI for the diagnosis of PDAC [19]. Its diagnostic reliability, however, can be limited by overlying bowel gas and patient body habitus [19]. Furthermore, while the addition of positron emission tomography (PET)/CT is currently not routinely recommended in PDAC patients [18], it may be considered in patients who are at high risk for the presence of distant metastases [17]. PET/ CT with ^68^Ga-labeled somatostatin analogs has a high sensitivity and specificity for tumor manifestations of non-insulinoma pancreatic neuroendocrine tumors (NETs) and is therefore recommended by the European Neuroendocrine Tumor Society (ENETS) Consensus Guidelines to fully stage the extent of disease in these patients [20].

While CT perfusion is not explicitly mentioned in the most widely used pancreatic tumor guidelines, both the National Comprehensive Cancer Network (NCCN) and American Society of Clinical Oncology (ASCO) Clinical Practice Guidelines consider contrast-enhanced CT as the preferred imaging modality for assessment of extent of disease in PDAC [17, 21]. According to the European Society for Medical Oncology (ESMO) Clinical Practice Guidelines, CT also is the basic radiological method for imaging of pancreatic NETs [22]. This allows for a convenient extension of the pancreatic CT protocol with CT perfusion imaging in difficult cases, e.g., tumors that are obscure on standard CT imaging.

A number of studies have been performed to find optimum study parameters for CT perfusion measurements, but have also shown changes in the quantitative results of CT perfusion measurements based on CT examination parameters and evaluation procedures, e.g., depending on the temporal sampling rate [23, 24], the image noise [25], the motion correction [26, 27], the mathematical perfusion model [28] and even the employed version of the post-processing software [29]. That is, based on the acquisition parameters and evaluation settings, different quantitative results can be expected for the same measurement, which limits the comparability of measurements between studies and in clinical practice. Thus, a standardization of acquisition protocols and evaluation procedures is necessary to achieve reliable and clinically meaningful measurements. This need has also been recognized by the Experimental Cancer Medicine Centre Imaging Network Group which published their guidelines for the assessment of tumor vascular support with dynamic contrast-enhanced computed tomography in 2012 [30].

This study aims to provide a comprehensive overview of the study parameters and evaluation procedures used in clinical CT perfusion studies of the pancreas by means of a quantitative meta-analysis. Furthermore, statistical analysis is performed to confirm the individual study results on the potential applications of perfusion CT as well as to identify the acquisition parameters with the strongest influence on quantitative results for further investigation and standardization.

## Methods

### Search strategy

We explored the PubMed library, searching the database for published studies with the key terms ‘pancreas’ or ‘pancreatic,’ ‘dynamic’ or ‘perfusion,’ and ‘computed tomography’ or ‘CT’. All CT perfusion research studies published and indexed before July 3, 2020, were collected. A total of 491 research articles published from 1982 to 2020 were obtained from the search. In a first step, these research articles were scanned manually based on their titles and abstracts for inclusion/exclusion. In a second step, all suitable studies were then read and assessed by two authors independently (N.V. and S.S.). Disagreements regarding study inclusion/exclusion were resolved by consensus-based discussions in two cases.

### Inclusion and exclusion criteria

We included studies that (1) reported original data, (2) included original human data, (3) included at least five or more datasets, (4) included measurements of the pancreas or pancreatic pathologies, and (5) reported quantitative perfusion parameters from perfusion CT. All study designs (prospective and retrospective) were included. Studies that had a cohort overlapping with previously published studies were not considered original data and excluded. Articles reporting different perfusion measurements for the same patient collective were counted as one study, since patient data were acquired only once [14, 31, 32]. Reviews, animal studies, or case reports (less than five patients) were excluded. To distinguish perfusion CT from conventional contrast-enhanced CT studies, only studies that reported quantitative perfusion parameters were included.

### Data extraction

Data were extracted from all the included research studies by two authors independently (N.V. and S.S.). Disagreements regarding data extraction were resolved by consensus-based discussions in five cases. Data extracted included study parameters and reported mean values of quantitative perfusion parameters (blood flow (BF), blood volume (BV), and permeability). Here, study parameters include (1) sample size, (2) CT examination parameters: acquisition parameters (tube potential, tube current–time product, anatomical coverage, total acquisition time, lowest temporal sampling, highest temporal sampling, and use of variable temporal sampling), reconstruction parameters (slice thickness), contrast agent information (amount of contrast agent, iodine concentration, total amount of iodine, injection rate, and use of saline flush), dose information (effective dose), and (3) post-processing information (perfusion model and type of post-processing software). Many studies used variable temporal sampling rates, e.g., a sampling rate of one acquisition per second for the first 30 s followed by one acquisition every 5 s for the next 60 s. Therefore, the lowest temporal sampling rate and highest temporal sampling rate used in each study were considered separately for analysis. All reported quantitative measurements from the studies were then grouped into four categories based on the clinical entities reported by the study: (1) normal pancreas, (2) pancreatitis, (3) PDAC, and (4) non-PDAC. Here, the category “normal pancreas” includes both patients without pancreatic pathology and measurements in non-pathologic tissue. The non-PDAC group includes all pathologies other than pancreatitis and PDAC, i.e., insulinoma or endocrine tumors, etc., which were grouped despite their physiological differences because of the low number of studies.

### Statistical analysis

Statistical analysis was performed using Microsoft Excel 2016 and SAS software (version 9.2, SAS Institute, Cary, N.C., USA). For all study parameters, histogram distribution plots were computed for qualitative analysis of parameter distributions, and medians and interquartile ranges were calculated. Year-wise means of number of studies, sample size and CT examination parameters were calculated and linear regression plots were computed using these mean values to analyze the trend of study parameters over the years. Mean values and standard deviations of reported perfusion values were calculated for each pathology. Weighted analysis of variances (W-ANOVA) followed by Student’s *t* test was performed for comparing reported perfusion values of different pancreatic pathologies to each other while weighting the measurements by the number of patients. Outliers have been removed from reported perfusion values based on a range of mean ± 1.96 * standard deviations of the parameter values, as some studies reported very high measurement values. W-ANCOVA was performed to test the effect of the individual CT examination parameters on reported perfusion values, simultaneously considering the effect of the clinical entities.

Qualitative box-plot analysis was performed for those CT acquisition parameters which showed a statistically significant effect on the quantitative measurements with respect to the pancreatic pathologies. To this end, quantitative measurements were separated into two groups based upon CT acquisition parameters (i.e., for each acquisition parameter a low-value and a high-value group), where the threshold value between the two groups was determined using *K*-means clustering algorithm, visualizing differences in quantitative measurements based on acquisition settings.

Total acquisition time and effective dose were compared between studies using single temporal sampling and studies using variable temporal sampling by Student’s *t* test.

## Results

**Fig. 1:**
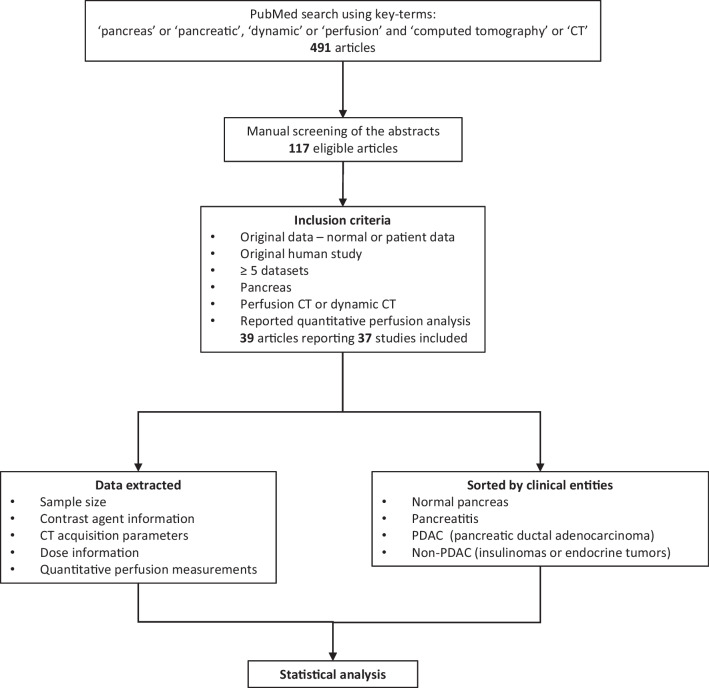
Flowchart illustrating the study design and the inclusion process

In total, 491 published research articles were collected for the current study. After manual screening of the title and abstracts, 117 articles were selected for full-text screening. Out of these, 39 articles reporting 37 studies were finally included in the current study based on the inclusion criteria [6, 7, 9, 10, 12–14, 28, 31–60]. A flowchart illustrating the selection of the studies is shown in Fig. 1. A summary of the information on the number of studies reporting each quantitative perfusion parameter (blood flow, blood volume, and permeability) for the respective clinical entity is shown in Table 1.

**Table 1:** Number of studies (number of patients) reporting the quantitative parameters (blood flow, blood volume, and permeability) for the respective clinical entity

Clinical entities	Perfusion parameters		
	Blood flow	Blood volume	Permeability
*Number of studies (Number of patients)*			
Normal pancreas	30 (983)	18 (707)	13 (448)
Pancreatitis	10 (281)	9 (270)	5 (111)
PDAC	20 (591)	14 (458)	10 (306)
Non-PDAC	4 (231)	5 (249)	3 (57)

*PDAC*, pancreatic ductal adenocarcinoma

**Fig. 2:**
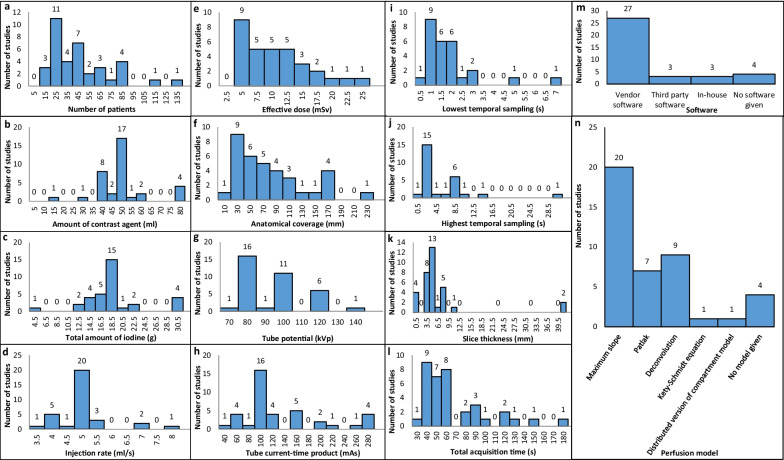
Histogram distribution of: **a** sample size (number of patients), **b**–**l** CT examination parameters, and (**m**) the software and (**n**) perfusion model used for calculating the perfusion measurements, respectively. Please note that values higher than the highest histogram bin were put into the highest histogram bin. The total count of studies in histogram (**n**) is greater than the total number of studies as some of the reported studies have used more than one perfusion model for the analysis (i.e., maximum slope model and Patlak analysis)

Figure 2a–l shows the histogram distribution of the study parameters in the included studies. For most of the parameters, a wide range of values have been reported over the years such as the number of patients with a median (interquartile range) of 36.0 (23.0–57.0), effective dose with a median of 8.8 (4.9–11.6) mSv, and CT acquisition parameters (anatomical coverage: 69.0 (29.4–105.0) mm, tube potential: 95.0 (80.0–100.0) kV_p_, tube current–time product: 100.0 (100.0–150.0) mAs and total acquisition time: 51.0 (40.0–79.5) s). By comparison, the contrast agent parameters show a considerable homogeneity and less variance around the median of 50.0 (40.0–50.0) mL of contrast agent, 17.5 (15.2–18.5) g of iodine, and 5.0 (5.0–5.0) mL/s of injection rate. The median of the lowest and highest temporal sampling rate was 1.5 (1.0–2.0) s and 2.0 (1.3–7.3) s, respectively, and median slice thickness was 5.0 (3.0–5.8) mm. Regarding the type of post-processing software, most of the research studies preferred to use vendor software as compared to third-party or in-house software. The most commonly used perfusion model for quantification of pancreatic perfusion parameters was the maximum slope model, followed by the deconvolution model.

**Fig. 3:**
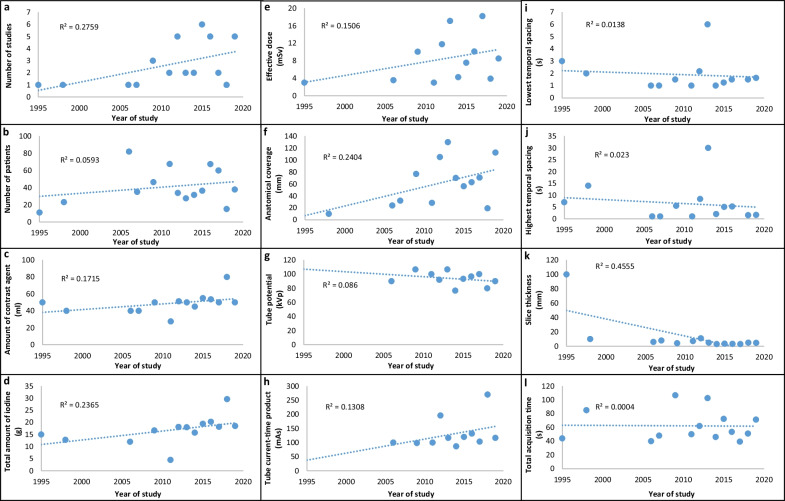
Linear regression plots of the means of (**a**) number of studies, (**b**) sample size (number of patients), and (**c**–**l**) all the CT examination parameters computed over the years. Please note that the plot for injection rate was omitted, as there was no observable trend of the mean injection rate

Linear regression plots of the mean values of number of studies, sample size and CT examination parameters over the years are shown in Fig. 3, visualizing changes in parameters over time. The number of studies as well as the number of patients per study have been increasing after 2010. The amount of contrast agent used and hence the amount of iodine also shows an increasing trend. There was no notable trend for injection rate. Mean tube potential and tube current–time product have slightly decreasing and increasing trends, respectively. The total time taken for the acquisition and the effective dose do not show any notable trend over the years, while the anatomical coverage shows an increasing trend. The slice thickness also decreased over the years with mean minimum value reported as 2.5 mm. The lowest temporal sampling shows a notable decrease over the years, i.e., shorter intervals between individual acquisitions, whereas the highest temporal sampling rate only shows a slight decrease.

**Fig. 4:**
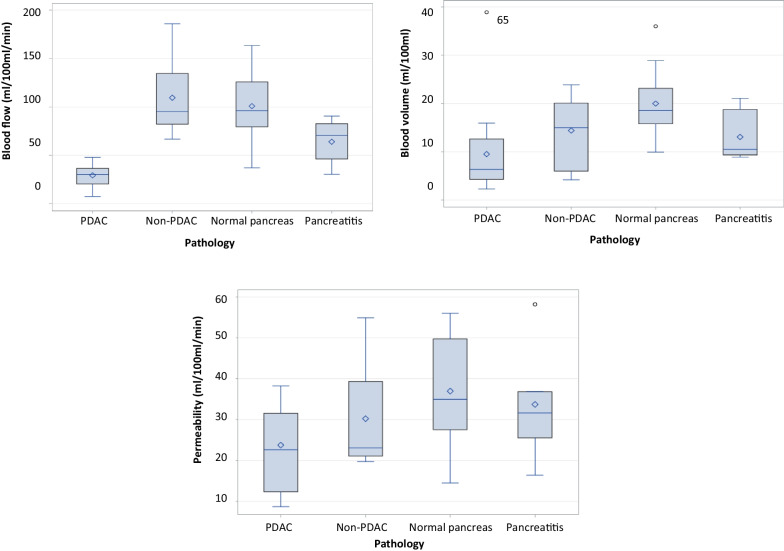
Boxplots for comparing the reported results of quantitative measurements of blood flow, blood volume, and permeability within the different pancreatic pathologies. *PDAC* = pancreatic ductal adenocarcinoma

**Fig. 5:**
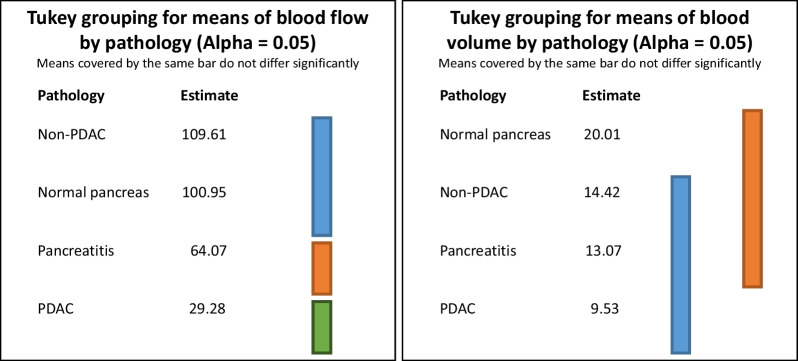
W-ANOVA Tukey grouping for comparing the weighted means of quantitative measurements (blood flow [mL/100 mL/min] and blood volume [mL/100 mL]) between the different pancreatic pathologies. Please note that no groupings are shown for permeability, as the W-ANOVA model was not significant for permeability (*p* = 0.11). *PDAC* = pancreatic ductal adenocarcinoma

Mean ± SD (standard deviation) of the quantitative parameters (blood flow, blood volume, and permeability) have been calculated for the respective clinical entities, and the results are shown in Table 2. The lowest averaged values for all parameters were observed in the PDAC group as compared to pancreatitis, normal pancreas and non-PDAC. While mean blood flow was highest in non-PDAC, mean blood volume and permeability were highest in normal pancreas. Figure 4 also shows similar results with the box-plot representations of the reported quantitative parameters (blood flow, blood volume, and permeability) within different pancreatic pathologies. The W-ANOVA Tukey groupings for comparing the means of quantitative measurements (blood flow and blood volume) between different pancreatic pathologies are shown in Fig. 5. The W-ANOVA results show that blood flow (*p* < 0.001) and blood volume (*p* < 0.001) differ significantly between pancreatic pathologies and normal pancreas but permeability does not (*p* = 0.11). Student’s *t* test following W-ANOVA shows significant differences between normal pancreas/pancreatitis, normal pancreas/PDAC, and PDAC/non-PDAC based on the perfusion parameters blood flow and blood volume, and between pancreatitis/PDAC, and pancreatitis/non-PDAC for blood flow only (all *p* < 0.05, respectively).

**Table 2:** Mean ± SD of the quantitative parameters (blood flow, blood volume, and permeability) for the respective clinical entity

Clinical entities	Perfusion parameters		
	Blood flow (mL/100 mL/min)	Blood volume (mL/100 mL)	Permeability (mL/100 mL/min)
Normal pancreas	100.9 ± 30.9	20.0 ± 6.0	36.9 ± 13.4
Pancreatitis	64.0 ± 22.4	13.1 ± 5.0	33.7 ± 15.6
PDAC	29.3 ± 10.9	9.5 ± 8.9	23.7 ± 10.8
Non-PDAC	109.6 ± 39.9	14.4 ± 7.7	30.2 ± 16.5

*PDAC*, pancreatic ductal adenocarcinoma

Table 3 shows how many of the studies reported statistically significant differences for the comparison of different pancreatic pathologies based on quantitative perfusion measurements compared to the total number of studies reporting results of statistical analysis for each parameter and for each comparison. Similar to the total number of studies shown in Table 1, most of the studies focused on PDAC, followed by pancreatitis, with only a limited number of results available for non-PDAC. With two exceptions, all the studies reporting blood flow and blood volume showed significant differences in their results. For studies analyzing permeability, only 50% have reported significant differences between the investigated clinical entities.

**Table 3:** Overview of the studies reporting significant statistical results for comparing the pancreatic pathologies based on perfusion measurements versus the studies reporting the perfusion parameters

Overview of statistical analysis (19 studies total)				
Studies reporting significant statistical results/Studies reporting perfusion values				
Compared clinical entities	Total studies combined	Blood flow	Blood volume	Permeability
Normal pancreas versus Pancreatitis	8/8	8/8	7/8	2/4
Normal pancreas versus PDAC	14/16	14/14	10/10	3/7
Normal pancreas versus Non-PDAC	3/3	2/3	2/3	0/2
Pancreatitis versus PDAC	3/4	3/3	3/3	2/3
Pancreatitis versus Non-PDAC	0	0	0	0
PDAC versus Non-PDAC	0	0	0	0

*PDAC*, pancreatic ductal adenocarcinoma

The effects of the CT examination parameters and perfusion model on the quantitative measurements are summarized in Table 4. The amount of contrast agent, injection rate, and use of saline flush show a significant effect on reported blood flow. Tube current–time product, highest temporal sampling, and use of variable temporal sampling, have a significant effect on reported blood volume. None of the evaluated parameters show a significant effect on reported permeability.

**Table 4:** Effects of the CT examination parameters and evaluation technique on the quantitative measurements with the obtained *p*-values stated in parentheses

	Blood flow	Blood volume	Permeability
*Effects on quantitative measurements*			
Amount of contrast agent	**✔ (0.0422)**	✘ (0.6681)	✘ (0.5636)
Amount of iodine	✘ (0.9605)	✘ (0.3591)	✘ (0.5063)
Total amount of iodine	✘ (0.0767)	✘ (0.7626)	✘ (0.5480)
Injection rate	**✔ (0.0150)**	✘ (0.4055)	✘ (0.3556)
Effective dose	✘ (0.7755)	✘ (0.1162)	✘ (0.3293)
Anatomical coverage	✘ (0.5885)	✘ (0.1600)	✘ (0.6122)
Tube potential	✘ (0.5329)	✘ (0.1386)	✘ (0.5088)
Tube current–time product	✘ (0.5742)	**✔ (0.0152)**	✘ (0.6112)
Lowest temporal sampling	✘ (0.2455)	✘ (0.1390)	✘ (0.9258)
Highest temporal sampling	✘ (0.0811)	**✔ (0.0348)**	✘ (0.6057)
Slice thickness	✘ (0.0960)	✘ (0.3996)	✘ (0.6851)
Total acquisition time	✘ (0.9281)	✘ (0.5947)	✘ (0.3858)
Use of variable temporal sampling	✘ (0.1041)	**✔ (0.0078)**	✘ (0.9196)
Use of saline flush	**✔ (0.0067)**	✘ (0.4341)	✘ (0.9693)
Perfusion model	✘ (0.1619)	✘ (0.2880)	✘ (0.3007)

Bold values indicate a significant effect of the respective parameters on the quantitative measurement whereas non-bold values indicate a non-significant effect

**Fig. 6:**
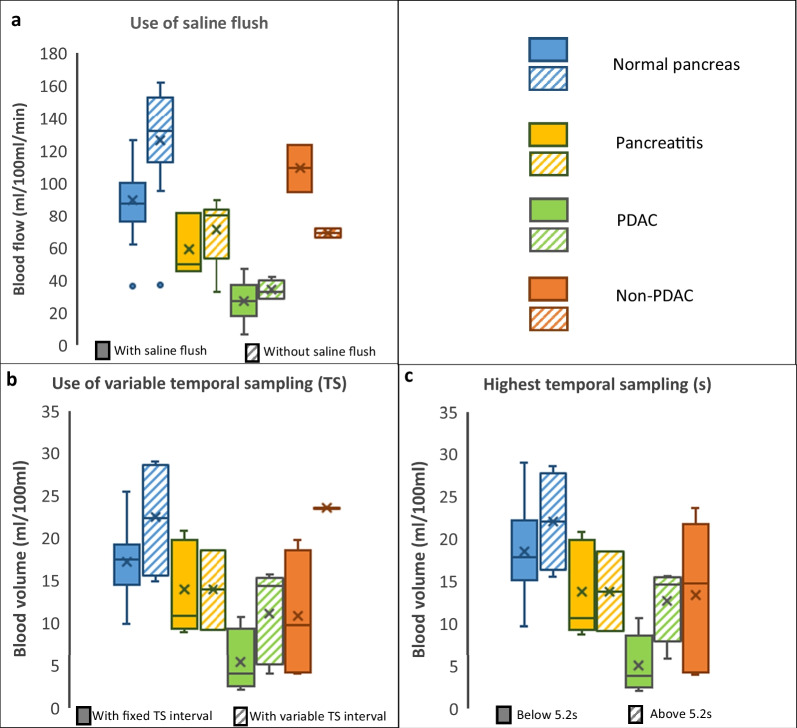
Boxplots comparing the effect of acquisition parameters on the quantitative measurements (blood flow and blood volume) within different pancreatic pathologies: **a**, **b** show the binary effect of saline flush and temporal sampling on the blood flow and blood volume, respectively; **c** shows the effect of highest temporal sampling on blood volume. Please note the differences in sample sizes between the groups (cf. Tab. 1). *PDAC* = pancreatic ductal adenocarcinoma

Figure 6 shows examples of box plots comparing the effect of CT examination parameters (which were reported as significant, cf. Table 4) on the quantitative measurements (blood flow and blood volume) within different pancreatic pathologies. These plots illustrate how the acquisition parameters influence the results, e.g., that higher values of blood flow were found in normal pancreas when no saline flush was used (mean blood flow 127.69 ± 37.21 mL/100 mL/min vs. 90.35 ± 20.97 mL/100 mL/min). Similarly, higher values of blood volume were found when using variable temporal sampling rates (mean blood volume 22.81 ± 5.68 mL/100 mL vs. 17.40 ± 4.37 mL/100 mL).

The difference in total acquisition time between studies with single temporal sampling rates and studies with variable temporal sampling rates was statistically significant (*p* = 0.03). Longer acquisition times were found for studies with variable temporal sampling rates. However, the difference in effective dose between studies with single temporal sampling rates and studies with variable temporal sampling rates was not statistically significant (*p* = 0.19).

## Discussion

The aim of this study was to perform a quantitative meta-analysis of pancreatic CT perfusion studies with regard to study parameters, the possibility to discriminate pancreatic diseases quantitatively based on perfusion measurements, and to investigate the connection between study parameters and quantitative measurements.

The results show significant differences of measured blood flow and blood volume between different pathologies as well as healthy tissue, validating the use of CT perfusion as a quantitative imaging biomarker despite the extremely heterogeneous nature of the dataset. This is in agreement with individual results of the evaluated studies, which reported significant differences in perfusion parameters when comparing different pathologies or pathologies to non-pathological tissue (cf. Table 3). However, further research is necessary regarding the value of measurements of permeability based on the results reported individually in the evaluated studies and this quantitative meta-analysis. Furthermore, significant differences between non-PDAC and normal pancreas reported in studies could not be reproduced by this quantitative meta-analysis [14, 39]. This can be explained by the heterogeneous definition of “non-PDAC” in this meta-analysis, which includes insulinomas and endocrine tumors, to compensate for the small number of available studies. Additionally, histopathological studies show an increased microvessel density (MVD) for PDAC over normal pancreas [61], while CT perfusion shows a decreased blood flow (cf. Fig. 4). One theory is that the increased fluid pressure in the tumor stroma leads to reduced blood flow through the compressed vessels despite high MVD [62], but further research is necessary.

Being a meta-analysis, the evaluation in the current study focused on differences between pathologies, which limits clinical applicability of the results. For example, the results show significant differences in blood flow between PDAC and pancreatitis. However, we did not perform sub-group analysis on mass-forming chronic pancreatitis, which can be difficult to distinguish from PDAC. This kind of specific clinical application falls outside of the scope of this meta-analysis, but individual studies show promising results for CT perfusion in this regard [50].

Study parameters in evaluated studies were very heterogeneous and changed over time, which highlights the need for standardization, if comparability between CT perfusion measurements performed at different institutions is to be achieved. This need for standardization is further highlighted by the statistical analysis showing a significant effect of multiple acquisition parameters on reported quantitative results. Furthermore, this analysis indicates which parameters might be most important for standardization, while the box-plot analysis provides a qualitative investigation of effect sizes. However, when interpreting these results, different sample sizes for parameters and pathologies have to be considered (cf. Table 1). Many studies did not fully comply with the reporting guidelines proposed by the Experimental Cancer Medicine Centre Imaging Network Group in 2012 [30], reporting only some acquisition parameters, further limiting sample sizes. In consequence, results regarding blood flow and normal pancreas should be considered most reliable when interpreting Table 3 and Fig. 6. While the current dataset does not allow to recommend specific acquisition settings, results are generally in line with previous studies, which agree on the importance of these acquisition parameters, e.g., temporal sampling rate [23], contrast agent injection protocols [36], and image noise [25]. The results also underline the value of variable temporal sampling, which can be used to reduce the sampling rate after the first-pass of the contrast agent, allowing for longer acquisition times without increasing radiation exposure. However, previous results on the influence of the perfusion model on quantitative measurements could not be reproduced [28]. This might be explained by the fact that many studies did not report the perfusion model and the wide range of models used (cf. Fig. 2).

The main limitation of this meta-analysis is the limited number of included studies, which is based on the number of available studies on the topic of pancreatic CT perfusion. Considering that not all of the evaluated parameters are reported in every study, the sample size is even more limited for some of the evaluations in the current study. The sample size was also even more limited for some of the investigated pathologies, which resulted in the pooling of all tumor entities that are not adenocarcinoma in a heterogeneous “non-PDAC” group. Similarly, some studies reported measurements for normal pancreatic tissue and pathological tissue from the same patients, but an effect of the investigated diseases on the normal tissue cannot be precluded, so a further distinction between patients with and without pancreatic disease might be necessary [61]. Based on the small sample size, multivariate statistical analysis was not possible, and only the detection and distinction of tumors/PDAC and pancreatitis was statistically validated in this study. Evaluating studies performed for a different organ, e.g., the brain, where a larger number of studies is available might yield further insights, but physiological differences between the organs and body regions have to be considered when interpreting and comparing results. In turn, results from this study cannot be directly transferred to perfusion studies of other organs and body regions. Furthermore, studies from a time frame of more than 25 years were included in this study. While CT technology advanced considerably during this time, reported perfusion values remain compatible with each other, and the statistical analysis reported no significant effect for many parameters indicative of technological progress, like anatomical coverage. Similarly, all study parameters from individual studies were included in the analysis as reported to avoid any bias toward certain sets of parameters. Finally, while this study highlights the study parameters with a significant influence on reported quantitative results, prospective studies are needed to determine optimum settings for those parameters.

In view of use in clinical practice, CT perfusion as well as MRI can be helpful for tumor detection when a suspected pancreatic tumor is not visible on routine pancreatic CT imaging [17]. Two small studies directly compared CT perfusion and diffusion-weighted (DW) MRI in PDAC patients and reported CT perfusion and (DW)-MRI parameters to be comparably applicable for differentiation of PDAC lesions and non-neoplastic pancreatic tissue [54, 63]. In addition, the performance of both PET/CT and CT perfusion was reported to exceed the performance of standard CT for insulinoma detection [64, 65]. However, studies which directly compare these imaging modalities are lacking.

Furthermore, a major limitation of standard pancreatic CT is its inability to reliably assess the response of PDAC lesions to (radio-)chemotherapy (RCT) since it cannot differentiate post-treatment fibroinflammatory changes from residual viable tumor tissue [66]. Here, DW-MRI and perfusion CT have both shown potential for prediction of histopathological response after RCT [60, 66], although no study compared these imaging modalities in this regard.

## Conclusions

In conclusion, this study shows the value of pancreatic CT perfusion in the differentiation of pancreatic diseases, as results from individual studies could be reproduced in the pooled dataset despite the heterogeneity in CT protocols between studies. Furthermore, the need for standardization of CT perfusion protocols is highlighted, as a significant influence of study parameters on reported quantitative results was observed.

## Data Availability

All data generated or analyzed during this study are included in this published article.

## Abbreviations

ASCO: American society of clinical oncology
BF: Blood flow
BV: Blood volume
CT: Computed tomography
DWI: Diffusion-weighted imaging
ENETS: European neuroendocrine tumor society
ESMO: European society for medical oncology
MRI: Magnetic resonance imaging
MVD: Microvessel density
NCCN: National comprehensive cancer network
NET: Neuroendocrine tumor
PDAC: Pancreatic ductal adenocarcinoma
PET: Positron emission tomography
RCT: (Radio-)chemotherapy
SD: Standard deviation
US: Ultrasound
W-ANCOVA: Weighted analysis of covariances
W-ANOVA: Weighted analysis of variances
